# Phosphatidylcholine restores neuronal plasticity of neural stem cells under inflammatory stress

**DOI:** 10.1038/s41598-021-02361-5

**Published:** 2021-11-24

**Authors:** Dario Magaquian, Susana Delgado Ocaña, Consuelo Perez, Claudia Banchio

**Affiliations:** Instituto de Biología Molecular y Celular de Rosario (IBR, CONICET) Ocampo y Esmeralda, Predio CONICET and Departamento de Ciencias Biológicas, Facultad de Ciencias Bioquímicas y Farmacéuticas, Universidad Nacional de Rosario, 2000 Rosario, Argentina

**Keywords:** Neuroscience, Cellular neuroscience, Neurogenesis

## Abstract

The balances between NSCs growth and differentiation, and between glial and neuronal differentiation play a key role in brain regeneration after any pathological conditions. It is well known that the nervous tissue shows a poor recovery after injury due to the factors present in the wounded microenvironment, particularly inflammatory factors, that prevent neuronal differentiation. Thus, it is essential to generate a favourable condition for NSCs and conduct them to differentiate towards functional neurons. Here, we show that neuroinflammation has no effect on NSCs proliferation but induces an aberrant neuronal differentiation that gives rise to dystrophic, non-functional neurons. This is perhaps the initial step of brain failure associated to many neurological disorders. Interestingly, we demonstrate that phosphatidylcholine (PtdCho)-enriched media enhances neuronal differentiation even under inflammatory stress by modifying the commitment of post-mitotic cells. The pro-neurogenic effect of PtdCho increases the population of healthy normal neurons. In addition, we provide evidences that this phospholipid ameliorates the damage of neurons and, in consequence, modulates neuronal plasticity. These results contribute to our understanding of NSCs behaviour under inflammatory conditions, opening up new venues to improve neurogenic capacity in the brain.

## Introduction

Despite its diverse presentation, inflammation is a common feature across several neuropathological processes and has been implicated as a critical mechanism responsible for the progression of neurodegenerative disorders including Parkinson’s disease, Alzheimer’s disease, multiple sclerosis^1–3^ as well as traumatic brain injury^4,5^ and stroke^2,6,7^. Neuroinflammation is considered a double-edged sword, with protective as well as detrimental effects on the nervous system, especially during repair and recovery. In response to different types of injuries that cause neurons and oligodendrocytes death, activated astrocytes and the resident immune-like glial cells, the microglia, proliferate and generate proinflammatory cytokines (such as IL-1, IL-6, IFN-γ and TNF-α), chemokines, prostaglandins, and free oxygen radicals, often leading to the development of cerebral damage, and promoting macrophages infiltration^8^. Both kinds of cells act as a host defence mechanism eliminating cellular debris and releasing inflammatory factors. These factors finally activate astrocytes, which proliferate and form the glia scar to define a dense limiting border between the healthy and damaged tissue. Two major niches of neural stem cells (NSCs) that support neurogenesis are in the subventricular zone and in the dentate gyrus of the hippocampus of the adult mammalian brain^9,10^. NSCs are multipotent self-renewing cells that have a regenerative potential because they can proliferate, migrate and differentiate into neurons, astrocytes or oligodendrocytes and thus, promote functional and structural repair of the injured tissue. Several studies have evidenced a cross-talk between immune modulators and NSCs fate^11–13^. In response to inflammatory reactions, it was shown that the glia scar could prevent tissue regeneration by NSCs^8^, and that LPS-induced neuroinflammation caused synapse loss by a mechanism dependent of microglia activation and IL-1β secretion^14^. In this scenario, understanding the NSCs response to these conditions and the mechanisms involved in the integration into the injured brain will be critical for the development of effective therapeutic strategies using stem cells.

We have previously demonstrated that phospholipids affect the fate of post-mitotic neural precursors; specifically, phosphatidylcholine (PtdCho) promotes neuronal differentiation at expenses of astroglial and unspecified precursors^15^. As the loss of neurons is the detrimental outcome of brain injuries and neurodegenerative diseases, we asked whether PtdCho could still favour neuronal differentiation under inflammatory conditions, and thus prevent or restore tissue damage in this context. By different approaches we have demonstrated that under pro-inflammatory culturing conditions there is an increase in neuronal differentiation that could support a renewal mechanism needed for tissue reparation. Strikingly, under the same conditions, neurons also display an aberrant morphology that could reflect the deleterious effect of neuroinflammation. Interestingly, addition of liposome of egg-source PtdCho further induces neuronal differentiation and also rescues the morphological and functional deficit by modulating neuronal plasticity.

## Results

### Effect of inflammatory stress on NSCs proliferation

**Figure 1:**
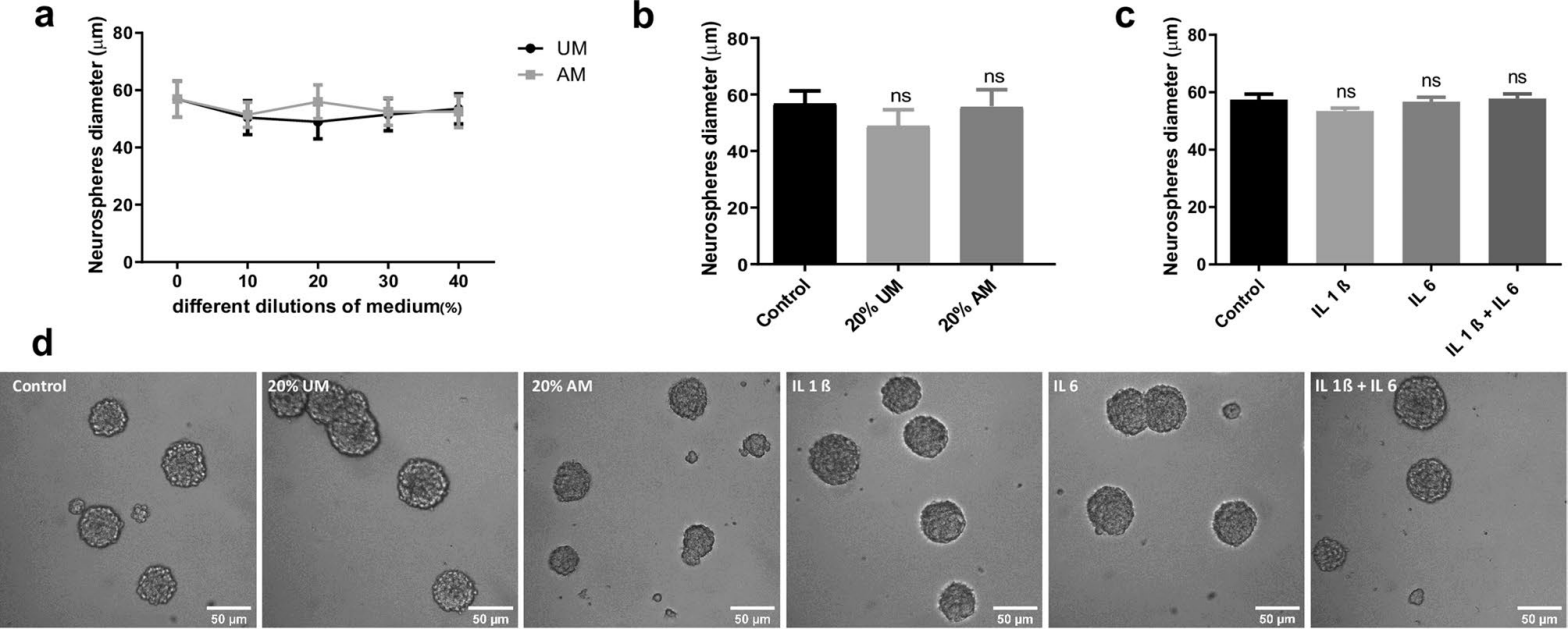
NSCs proliferation is not affected by inflammation. (**a**) After incubating the NSCs with different dilutions of medium obtained from LPS-stimulated macrophages (AM) or without stimulation as a control (UM) during 96 h, proliferation was analysed by measuring Neurosphere’s diameter. Graph represents the neurosphere’s diameter measured in three independent experiments. (**b**) Diameter of the Neurospheres of NSCs exposed to 20% V/V of UM and AM or control during 4 days. Graph represents the neurosphere’s diameter measured in three independent experiments. *ns* no statistical significance. (**c**) Diameter of the Neurospheres of NSCs exposed to 50 ng/ml of IL-1β and/or IL-6 during 96 h. Graph represents the neurosphere’s diameter measured in three independent experiments. *ns* no statistical significance. (**d**) Representative images (× 20) of neurospheres incubated in the indicated conditions. Scale bars: 50 μm.

The balance between NSCs proliferation and differentiation is essential for tissue repair^16^ and up to know is not clear how it is affected by inflammation. To that end, we incubated NSCs under normal proliferative condition (in the presence of EGF and FGF, neurosphere culture) supplemented with different concentrations (% V/V) of macrophages-activated media (AM) or with media without activation as a control (UM). We confirmed by RT-PCR that macrophages activated with LPS express and, as a consequence, secrete IL-1β, IL-6 and TNF-α to the media as previously demonstrated^17,18^ (Supplementary Fig. 1A). Treated cells were incubated for 4 days, and after this time, cell viability was analysed by MTT assay; UM plus LPS was also included to evaluate LPS toxicity as control (Supplementary Fig. 1B,C). To induce a moderate stress, cells were treated with AM (20% V/V) and, after 4 days, the neurosphere’s diameter was measured as a growth parameter (Fig. 1b). As Fig. 1a,b,d show, there is no significant change in the proliferation rate in the presence of different concentrations (V/V) of activated media respect to media without activation and control. To evaluate the role of each IL individually, similar analyses were performed in UM supplemented with IL-1β and/or IL-6. The concentrations were determined by assaying cell viability by MTT (Supplementary Fig. 1D). As Fig. 1c,d show, these ILs, as components of the inflammatory condition, do not affect the capacity of NSCs to proliferate as no changes were detected in the neurophere’s diameter under the assayed conditions.

### Effect of inflammatory stress on neuronal differentiation of NSCs

**Figure 2:**
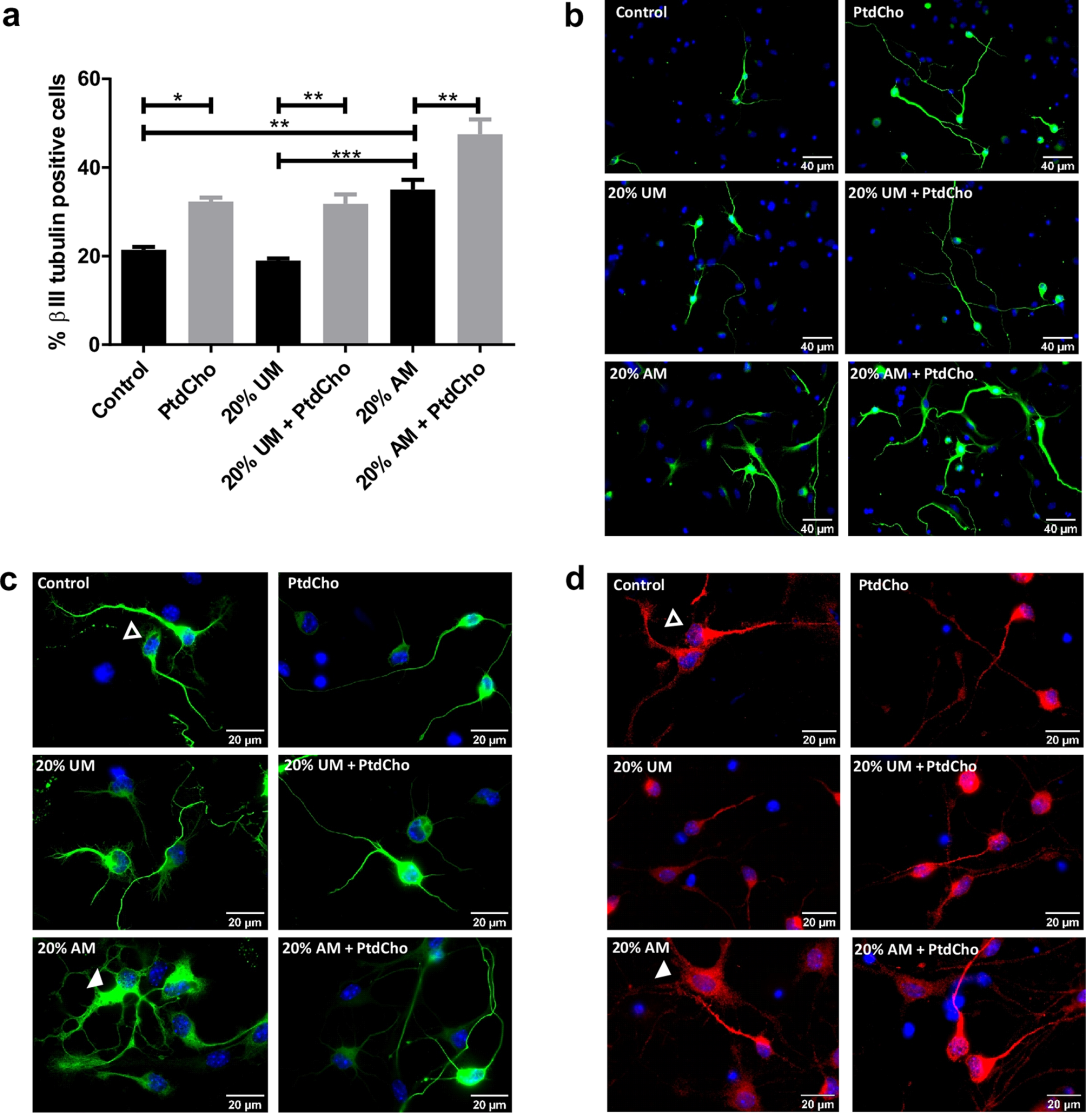
NSCs differentiation is affected by inflammation and restored by PtdCho. (**a**) Percentage of β-III tubulin positive cells analysed by immunocytochemistry coupled to fluorescence microscopy of NSCs exposed to 20% V/V of AM and UM in the presence or in the absence of PtdCho (50 μM) during 3 days. Graph represents the percentage of neuronal differentiation measured in five independent experiments. Data were presented as mean ± SEM. ***p < 0.001; **p < 0.01; *p < 0.05. (**b**) Representative images (× 40) of the immunofluorescence assays with the neuronal marker (β-III tubulin, green) nuclei (DAPI, blue). Scale bars: 40 μm. Representative images of neurons morphology under the indicated condition (× 100) βIII-tubulin (green) (**c**) or MAP2 (red) (**d**) Open arrow indicates normal neuron and white arrow indicates dystrophic neuron. Scale bars: 20 μm.

**Figure 3:**
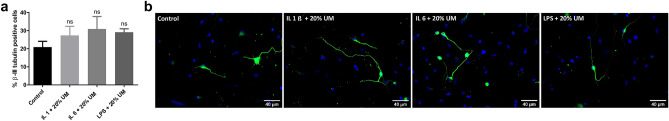
NSCs differentiation is not affected by ILs. (**a**) Percentage of β-III tubulin positive cells analysed by immunocytochemistry coupled to fluorescence microscopy of NSCs exposed 3 days to UM (20% V/V) supplemented with the indicated ILs (50 ng/ml) or LPS (1 µg/ml). Graph represents the percentage of neuronal differentiation measured in three independent experiments. *Ns* no statistical significance. (**b**) Representative images (× 40) of the immunofluorescence assays with the neuronal marker (β-III tubulin, green) and nuclei (DAPI, blue). Scale bars: 40 μm.

**Figure 4:**
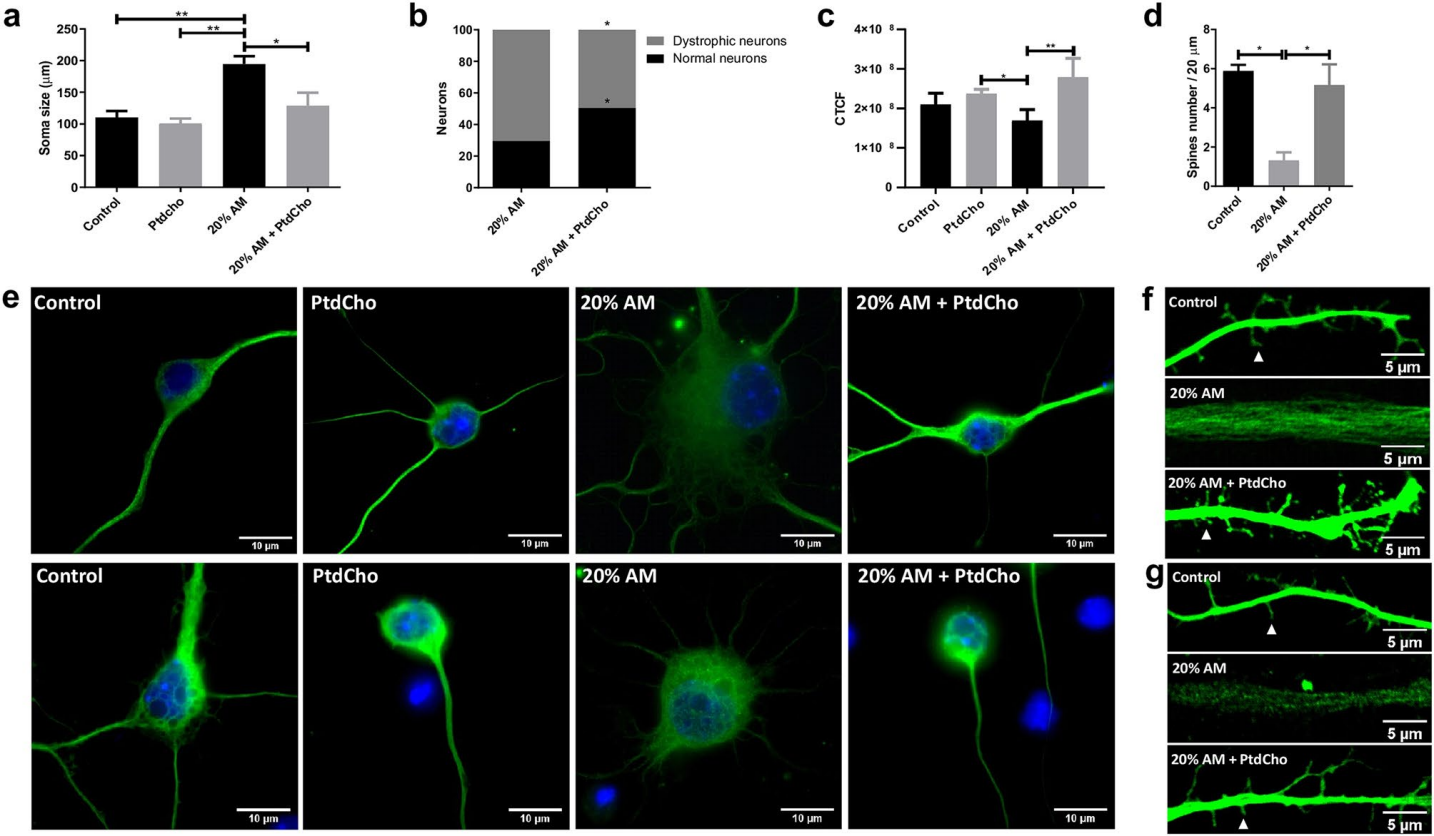
PtdCho rescues neuronal function induced by AM. (**a**) Quantification of neuron’s soma of cells incubated under the indicated condition. Graph represents the area of neuronal soma measured in four independent experiments. Data were presented as mean ± SEM. **p < 0.01; *p < 0.05. (**b**) Percentage of normal neurons (black bars) and dystrophic neurons (grey bars) after 3 days in culture under the indicated conditions *p < 0.05 (Student’s T-test). (**c**) βIII-tubulin signal expressed as corrected total cell fluorescence (CTCF). (**d**) Quantification of spine number. Values of quantification are expressed as mean ± SEM, *p < 0.05. (**e**) Representative images of neurons (× 100) Scale bars: 10 μm and dendritic spines (× 63) under the indicated conditions after 3 days (**f**) and 7 days (**g**) of culture. Arrowheads indicate spine. Scale bars 5 μm.

Neuronal differentiation is key in neural tissue regeneration after injuries^19^. To investigate this process under pro-inflammatory conditions, we analysed neuronal differentiation by immunocytochemistry using βIII-tubulin and MAP2 as neuronal lineage markers (Supplementary Fig. 2C). After culturing NSCs as neurospheres (two passages of 7 days each), cells were incubated for 3 days in media supplemented with macrophages-activated media (AM-20% V/V) as a condition of moderate inflammatory damage (Supplementary Fig. 1C), media without LPS activation (UM-20% V/V) or control media. The quantification analysis demonstrated that in the presence of AM there is a significant increase in the percentage of βIII-tubulin positive cells in comparison with the UM or the control (Fig. 2a,b). However, no changes were observed in astrocyte differentiation (data not shown). Similar results were observed at shorter time points (Supplementary Fig. 2A,B). However, a morphological observation revealed that neurons incubated with AM display a different shape than neurons at the control or neurons incubated with UM, with apparent tubulin disorganization, dystrophy with increased soma size, presence of vacuoles and poor development of dendritic spines (Fig. 2c,d, 4)^20,21^. These cells expressed neuronal markers βIII-tubulin and MAP2 and are negative for the astrocyte marker GPAF (Fig. 2c,d and Supplementary Fig. 2D). We therefore hypothesized that neuronal differentiation could be aberrant under pro-inflammatory stress, leading to cell dystrophy. To determine whether the observed effect is a direct action of LPS or the inflammatory components (IL-1β and IL-6), we evaluated neuronal differentiation in the presence or absence of each molecule individually. As Fig. 3 shows, treatment with UM supplemented with the indicated concentration of LPS, IL-1β and/or IL-6 did not affect the rate of neuronal differentiation nor the morphology of the neurons.

### Phosphatidylcholine enhances neuronal differentiation and ameliorates neuronal alterations caused by inflammatory conditions

We have previously demonstrated that PtdCho, as liposomes supplemented in the media, regulates the fate of post-mitotic precursor cells, inducing neurogenesis^15^. To test the effect of this molecule under pro-inflammatory stress conditions, we incubated NSCs under each condition in the presence of egg-source PtdCho (50 µM) and counted the resulting βIII-tubulin expressing cells. As shown in Fig. 2a,b, the pro-neurogenic effect of PtdCho shown in normal and UM control conditions, is also observed under inflammation, reaching the highest levels of neuronal differentiation. Interestingly, the aberrant phenotype observed in AM was ameliorated when cells were incubated in the presence of PtdCho (Fig. 2c,d, right panel). A detailed morphometric analysis performed after 3 and 7 days, demonstrated that the soma size, the level of βIII-tubulin expression and the presence of dendritic spines that have long been consider to provide the morphological bases for synaptic plasticity are recovered after PtdCho treatment (Fig. 4). More interestingly, the percentage of dystrophic neurons, decreases with PtdCho treatment, resulting in a significant increase in the phenotypically normal neurons (Fig. 4b) and suggesting that under inflammatory conditions, PtdCho not only regulates the fate of post-mitotic cells increasing neuronal differentiation^15^, but also rescues the dystrophic neurons, turning its morphology back to the normal.

### PtdCho restores synaptic defect caused by inflammation

**Figure 5:**
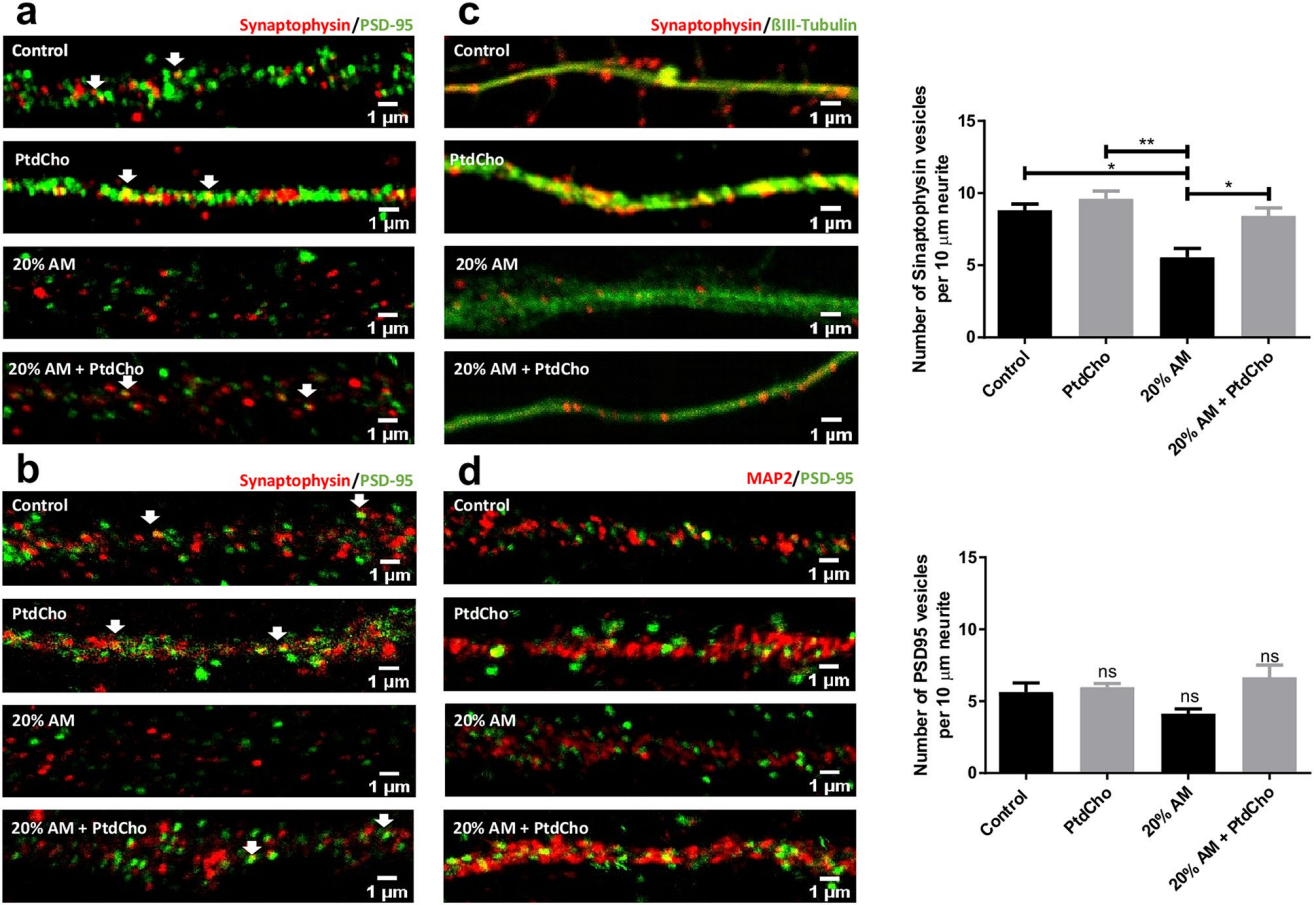
PtdCho supplementation restores synaptic defects caused by AM. Representative images (× 63) of the immunofluorescence assays with Synaptophysin (red) and PSD-95 (green) taken after 3 (**a**) and 7 (**b**) days of incubation in the indicated conditions. Scale bars: 1 μm. (**c**) Representative images (× 63) of the immunofluorescence assays with the neuronal marker (β-III tubulin, green) and synaptic vesicle marker (Synaptophysin, red). Scale bars: 1 μm. Quantification of Synaptophysin vesicles per 10 μm of neurite length. Graph represents the number of Synaptophysin-containing vesicles in 10 µm length measured in four independent experiments. Data were presented as mean ± SEM. **p < 0.01; *p < 0.05. (**d**) Representative images (× 63) of the immunofluorescence assays with the neuronal marker (MAP-2, green) and synaptic vesicle marker (PSD-95, red). Scale bars: 1 μm. Quantification of PSD-95 vesicles per 10 μm of length. Graph represents the number of PSD-95-containing vesicles in 10 µm length measured in four independent experiments. Data were presented as mean ± SEM. ns, no statistical significance.

We next examined whether the observed defect in neuronal morphology and in the formation of spines are associated with alteration in the development of structures related with the acquisition of neuronal functionality. We observed that AM induces a decrease on the synaptic puncta along dendrites after 3 and 7 days (Fig. 5a,b). Given the inflammation effect on synaptic clustering, and considering that the amount and localization of Synaptophysin and PSD-95 proteins play a critical role in synapse formation^22,23^, exo-endocytosis of synaptic vesicles^24^, neural plasticity^25,26^, memory^27^, motor development, behavioural features and cognitive impairments^28^, we then examined the expression of these synaptic proteins. Cells were immunoassayed for the presynaptic protein Synaptophysin and βIII-tubulin or the post-synaptic protein PSD-95 and MAP-2, and the relative number of signal puncta on neurites was counted and analysed by western blot. According with the dystrophic morphology, incubation with AM clearly and significantly decreases the number of Synaptophysin containing vesicles (Fig. 5c) relative to the control, but did not affect PSD-95 positive vesicles (Fig. 5d). Interestingly, incubation with PtdCho under inflammatory condition restores the number and the expression of Synaptophysin back to the control (Fig. 5c and Supplementary Fig. 3).

**Figure 6:**
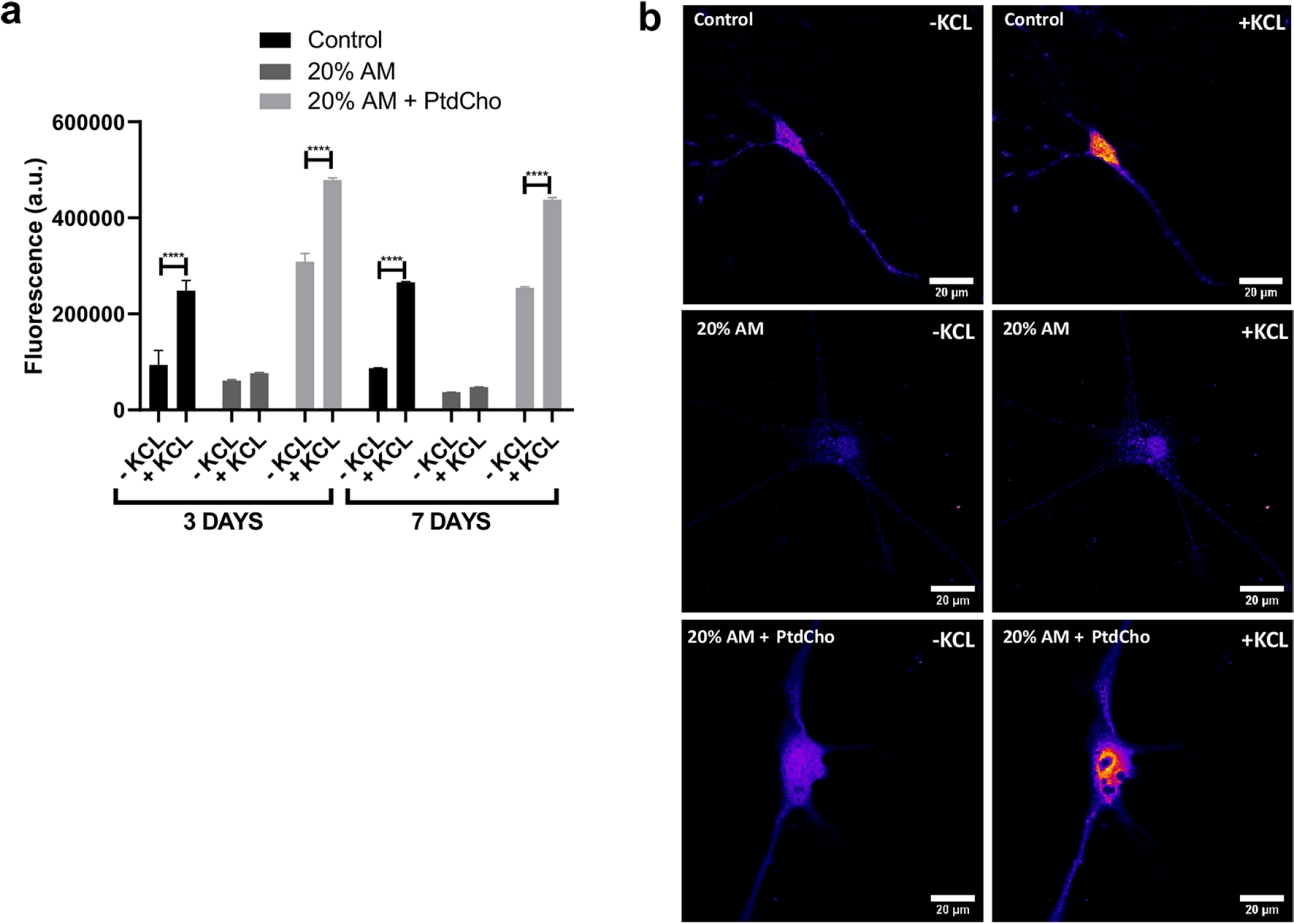
Stress affects neurons depolarization. (**a**) Cells were incubated under the indicated condition during 3 or 7 days and loaded with Fluo-3AM. Graph represents fluorescence as arbitrary units (a.u.) detected before and after treatment with KCl. Values are expressed as mean ± SEM ****p < 0.0001. (**b**) Representative images (× 63) of neurons labelled with Flio-3AM before and after KCl treatment. Scale bar 20 µm.

Depolarization-dependent calcium influx is an intrinsic property of synaptic vesicles, occurring in both mature and developing neuronal processes^29–31^. To determine whether neuronal cells incubated under stress of inflammation undergo depolarization, we measured intracellular Ca^2+^ levels by loading with Fluo-3AM cells differentiated during 3 and 7 days. As Fig. 6 illustrates, cells incubated under control condition show a rapid calcium influx in response to KCl. However, this capacity is negatively affected under inflammation, as no changes in fluorescence were detected (Fig. 6 and Supplementary video). Interestingly, when cells incubated under stress for 3 and 7 days were treated with liposome of PtdCho, this functional parameter was recovered (Fig. 6 and Supplementary video). This result clearly suggest that PtdCho has two effects: induces neurogenesis of NSCs and improves function of sick/damaged neuronal cells.

**Figure 7:**
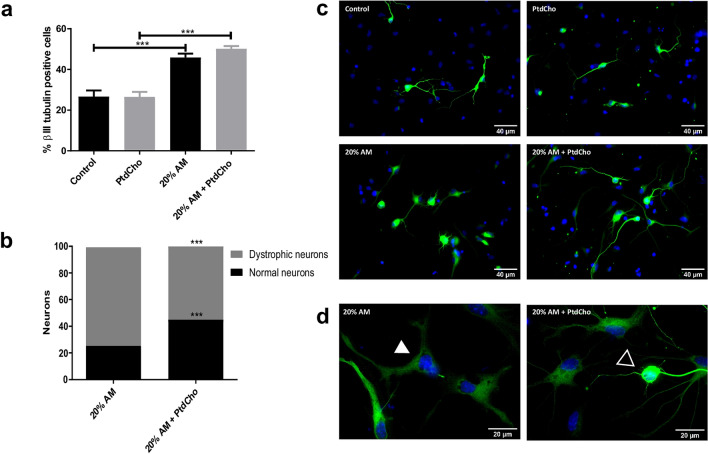
PtdCho restores morphology of dystrophic neurons. (**a**) Percentage of βIII-tubulin positive cells analysed by immunocytochemistry coupled to fluorescence microscopy of NSCs exposed to 20% V/V of AM when PtdCho was added later on, after 1 day of culture, and incubated for 3 days. Graph represents the percentage of neuronal differentiation measured in three independent experiments. Data were presented as mean ± SEM. ***p < 0.001. (**b**) Percentage of normal neurons (black bars) and dystrophic neurons (grey bars) after 3 days in culture under the indicated conditions. ***p < 0.001 (Student’s T-test). (**c**) Representative images (× 40) of neurons incubated under the indicated conditions Scale bars: 40 μm. (**d**) Representative images of neurons morphology under the indicated condition (× 100). Open arrow indicates normal neuron and white arrow indicates dystrophic neuron. Scale bars: 20 μm.

We have previously demonstrated that lipid treatment 1 day after plating the cells did not affect neuronal differentiation, indicating a narrow time-window of response in post-mitotic cells^15^. To confirm the effect of PtdCho independent of the promotion of neurogenesis, we quantified the percentage of neurons and the morphology adding PtdCho 1 day post inflammatory condition. As expected, PtdCho does not increase the percentage of neurons (Fig. 7a)^15^, but clearly altered the balance between healthy/normal neurons and dystrophic, pushing it to the normal population (Fig. 7b).

## Discussion

NSCs have a fundamental role after nervous tissue damage as they have the potential for regeneration owing to their capacity of self-renewal and differentiation into neurons^8,32^. However, this extraordinary capacity is limited under pathological conditions due to the factors present in the wounded microenvironment that can affect NSCs survival, proliferation and differentiation^33–36^.

Considering the difficulty of inducing NSCs to differentiate into nonglial cell types under stress condition, in this report, we provide details of the NSCs behaviour under inflammatory condition, a common scenario of many acute and chronic brain diseases. Furthermore, we provide evidence that PtdCho treatment could target NSCs conducting them towards functional neurons and also restoring the morphological deficit caused by inflammation.

Neuroinflammation can either affect the niche or the NSCs directly, with the end result of altered NSCs proliferation and/or differentiation^1,37^. We demonstrated that after 4 days of incubation of NSCs with AM or with individual cytokines, cell viability (Supplementary Fig. 1) and the rate of NSCs proliferation are not affected (Fig. 1). These results differ from previous demonstrating that proinflammatory cytokines reduce the number of new born neurons in the dentate gyrus in adult mice due to the restrain of the cell cycle^38^. As NSCs proliferation depends on the cell progression, we assumed that under our study condition cell cycle progression is not affected. In addition, the observed discrepancy could base on the different origin of the NSCs utilized.

The in deep study of the cellular mechanism leading to neuronal dysfunction under inflammatory condition is essential for the development of novel therapies. These experiments demonstrated that incubation of NSCs with 20% V/V of AM, but not with ILs individually (Fig. 3), induces aberrant neuronal differentiation, that give rise to dystrophic neurons (Fig. 2). The relatively constant number of neurons in AM-treated cultures during different periods of times (day 1 to day 3–7) suggests that AM does not affect specific step of neuronal differentiation process. Rather, it seems to be a very dynamic sequence of morphological changes with a constant progression to dystrophic morphology (Fig. 2 and Supplementary Fig. 2). It is well known that LPS activates microglia, and the consequent ILs secretion affects neuronal differentiation^39–41^ , we discard this effect as ILs and LPS alone did not affect neuronal differentiation of NSCs, nor the morphology of the neurons (Fig. 3).

Currently, lipids are taking a leading role in the nervous system. They have been shown to intervene in cellular functions such as proliferation, differentiation, cell cycle and act as pro-resolution lipid mediators in inflammatory events^15,42–45^. As this maladaptive neuronal plasticity that takes place under inflammation could be the reason of many brain failures, we evaluated the effect of PtdCho on NSCs differentiation. We demonstrated that PtdCho induces NSC differentiation toward neuronal lineage (βIII-tubulin and MAP2 positive cells) under inflammatory condition increasing the percentage of healthy non-dystrophic neurons (Figs. 2 and 4). Therefore, PtdCho changes the fate of post-mitotic cells increasing neurogenesis by turning on the PKA/CREB signalling pathway even under inflammatory conditions (AM). In fact, the percentage of βIII-tubulin positive cells decrease in the presence of PKA inhibitor (KT5720) (Supplementary Fig. 4). This specific effect of PtdCho could favour and increase the replacement of damaged neurons favouring NSCs-dependant neurogenesis. More interestingly, this phospholipid ameliorates the damage of neurons and, in consequence, modulates neuronal plasticity: in fact, treatment with PtdCho reinstitutes the morphology and also the levels of dendritic spines, the expression of Synaptophysin and synapsis clusters, and also the capacity of depolarization dependant calcium influx (Figs. 4, 5 and 6). This effect takes place even 1 day post inflammatory condition, hence, it decreases the amount of dystrophic neurons by a mechanism independent of PKA activity and NSCs differentiation (Fig. 7 and Supplementary Fig. 4). This capacity to modulate neuronal plasticity was also described for choline in the treatment of Rett Syndrome^46^.

In this stage, a repeated question arises: is choline^46^, CDP-Choline^47^ or PtdCho^15^ the key molecule for neuronal plasticity? Even though, choline has three main contributory roles in maintaining the cellular physiology in neurons: as precursor for the synthesis of the neurotransmitter acetylcholine^48^; as a key donor for methylation of DNA and regulation of gene expression^49^; and for the synthesis of PtdCho^50^, we propose that choline and CDP-Choline regulate the fate of NSCs and induces neurogenesis by its conversion into PtdCho. Experiments done in the presence of Hemicholineum-3 (a choline kinase inhibitor)^51^ or Edelfosine^45^ demonstrated that choline is unable to promote neuronal differentiation when the Kennedy pathway was blocked (Supplementary Fig. 5); however, PtdCho activates neurogenesis even under inhibition of these enzymes. This results clearly demonstrate that choline has to be converted in PtdCho to affect the fate of NSCs.

In conclusion, more research needs to be done to understand the molecular mechanism of PtdCho as modulator of neuronal plasticity, but, considering that loss and damage of neurons are the major consequence of acute and chronic neuroinflammation, these results might open a door to develop new therapeutic approaches.

## Methods

### Chemicals and antibodies

Dulbecco’s modified medium/Ham’s F12 (DMEM/F12 1:1), Dulbecco's Modified Eagle's Medium (DMEM), B27 and anti-rabbit Alexa Fluor® 488-labeled were purchased from Life Technologies Corporation (Carlsbad, CA, USA). Fetal bovine serum (FBS) from Internegocios (Buenos Aires, Argentina). Rabbit anti-β-Tubulin III antibody from Sigma (St. Louis, MO, USA), mouse anti-synaptophysin, mouse anti-MAP2, mousse anti- β-Actin and KT5720^52^ from Santa Cruz (Dallas, Texas, USA), rabbit anti-PSD-95 (Invitrogen) and anti-mouse Cy3-labeled from Millipore (Massachusetts, USA). Quick-Zol from Kalium (Buenos Aires, Argentina). Fluo-3/AM from Biotium (Landing Parkway Fremont, CA, USA), RNase-free RQ1 DNase enzyme, Reverse Transcriptase enzyme M-MLV from Promega (Wisconsin, USA). TAQ polymerase buffer, dNTPs and TAQ polymerase from TransGen Biotech (Beijing, China). Protease inhibitor cocktail, poly-d-lysine (PDL), epidermal growth factor (EGF), human basic fibroblast growth factor (bFGF), lipopolysaccharide (LPS) and Phosphatidylcholine (P3556) from egg yolk source were from Sigma (St. Louis, MO, USA). As specified in product information, they have a purity over 99% and a fatty acid content of approximately 33% palmitic, 13% stearic, 31% oleic, and 15% linoleic. In addition the detailed fatty acid composition of the mixture of egg yolk phosphatidylcholine and phosphatidylethanolamine has been recently described^53,54^.

### Animal studies and fetal neural stem cells culture

All animal experiments and related experimental protocols were approved by the Bioethics Commission for the Management and Use of Laboratory Animals of National University of Rosario, Argentina (N 6060/89). All procedures were carried out in accordance with the approved guidelines (Guide for the care and use of Laboratory Animals- 8° edition- e National Academies Press-Washington DC 2011 and in compliance with the ARRIVE guidelines). Time pregnant female C57/BL6 mice (gestation day 13) were sacrificed by cervical dislocation under supervision of the Animal Care and Use Committee. Neurospheres were obtained from E13 cortical cells as previously described^55^. Briefly, the lateral portion of the dorsal telencephalon (cortex) of embryonic day 13 mouse C57/BL6 was isolated. The cortices were chemically disrupted adding trypsin (0.05% w/v) for 5 min and then mechanically disrupted into single cells by repeated pipetting in medium DMEM/F12 (1:1) containing 10% FBS, penicillin G (100 units/ml) and streptomycin (100 μg/ml). Cells were centrifugated at 1000 rpm for 5 min and the pellet resuspended in serum-free medium DMEM/F12 (1:1). Dissociated cells were cultured at a density of 5 × 10^4^ cells/ml in medium DMEM/F12 (1:1) supplemented with B27, 10 ng/ml bFGF and 10 ng/ml EGF, at 37 °C in a humidified 5% CO_2_ incubator. Within 7 days, cells grew as free coating neurospheres that were then collected by centrifugation, and chemically and mechanically dissociated to obtain a new passage. These cells were incubated in the same media for another 7 days to ensure a homogeneous NSCs culture. For cell differentiation, neurospheres from the second passage were chemically and mechanically dissociated. After counting, 2.5 10^5^ cells were plated on poly-d-lysine (PDL) (10 μg/ml)-coated 24 well plates, or 5 × 10^4^ cells were plated on PDL (10 μg/ml)-coated 96 well plates in medium DMEM/F12 (1:1) supplemented with B27.

### Macrophages culture and LPS-induced stimulation

The mouse cell line Raw 264.7 (ATCC® TIB-71™) was cultured in DMEM 10% FBS supplemented with penicillin G (100 units/ml), streptomycin (100 μg/ml) (proliferation conditions) and maintained in a 5% CO_2_ humidified incubator at 37 °C. For activation, cells were grown to 80% confluence in petri dishes with DMEM medium supplemented with 10% FBS. At this time, cells were centrifuged at 1500 rpm for 10 min. The cell pellet was resuspended in 1 ml of DMEM/F12 medium and cells were transferred to a new plate containing DMEM/F12 stem cell medium (in the absence of FBS, B-27 and growth factors). For stimulation, pure LPS was added in a final concentration of 1 µg/ml. After 18 h of incubation, cells were centrifugated at 1000 rpm for 5 min and the culture medium was filtered through 0.22 μm filters (Sartorius) and stored immediately at − 80 °C.

### Total RNA isolation, DNase treatment and retrotranscription reaction

Murine macrophage RAW 264.7 total RNA was extracted in Quick-Zol (Kalium) following the supplier's specifications. Briefly, cells were resuspended in 1 ml Quick-Zol and incubated for 5 min at room temperature. Then, 0.2 ml of chloroform was added and they were centrifuged at 12,000 rpm for 10 min at 4 °C. Then, the aqueous phase was transferred to a new tube, 0.5 ml of isopropanol was added and the samples were incubated for 24 h at – 20 °C. The following day, they were centrifuged at 12,000 rpm for 10 min at 4 °C, the pellet was washed with 75% ethanol and centrifuged again at 12,000 rpm for 5 min at 4 °C. To evaluate the quality and quantity of the RNA obtained, absorbance measurements were made at 230, 260 and 280 nm (NanoVue Plus, General Electrics). Next, 1 µg of RNA was seeded on a 1.8% agarose gel to evaluate its integrity**.** To remove DNA from the samples, 10 μg of the RNA/DNA mixture was treated for 2 h with the RNase-free RQ1 DNase enzyme (Promega) at 37 °C. The reaction was then stopped by incubating the sample at 65 °C for 10 min. Once the treatment was completed, the RNA concentration was determined spectrophotometrically (NanoVue Plus, General Electrics). For the reverse transcription reaction, 2 μg of RNA was treated with the Reverse Transcriptase enzyme M-MLV (Promega) strictly following manufacturer’s instructions. For a final volume of 25 μl: oligo dT 20 ng/μl, dNTPs 0.5 mM each, buffer M-MLV 5 X, RNase inhibitor 0.8 U/μl and M-MLV Reverse Transcriptase 8 U/μl were added.

### Polymerase chain reaction (PCR)

The PCR reactions were performed in the following buffer for a final volume of 50 μl: 1 × TAQ polymerase buffer, 3 mM MgCl_2_, 25 μM dNTPs each, 0.4 pmol/μl oligonucleotides each, 0.2 U/μl of TAQ polymerase (Easy TAQ, TransGen Biotech). Gene Amp (Parkin-Elmer, Shelton, CT, USA) or My Cycler (BioRad, USA) thermal cyclers were used. The oligonucleotides sequences (5′–3′) used are: IL-1β Forward TTCAGGCAGGCAGTATCACTC, IL-1β Reverse GAAGGTCCACGGGAAAGACAC; IL-6 Forward TAGTCCTTCCTACCCCAATTTCC, IL-6 Reverse TTGGTCCTTAGCCACTCCTTC; TNF-α Forward CCTGTAGCCCACGTCGTAG, TNF-α Reverse GGGAGTAGACAAGGTACAACCC. The amplification reaction started with an initial denaturation for 10 min at 94 °C followed by 35 cycles of denaturation at 94 °C for 1 min, annealing at 60 °C for 30 s and elongation at 72 °C for 30 s. The last cycle was followed by a 10-min extension step at 72 °C. The amplified products were analyzed by ethidium bromide-stained agarose gel electrophoresis.

### Cell viability and proliferation assays

Cell viability was assessed by MTT-reduction assay. After cell treatment, MTT (5 mg/ml) was added to the cell culture medium at a final concentration of 0.5 mg/ml and incubated for 4 h at 37 °C, 5% CO_2_. The assay was stopped by replacing the MTT-containing medium with DMSO. The extent of MTT reduction was measured spectrophotometrically at 570 nm^56^. Results are expressed as a percentage of the control.

Proliferation of NSCs was assayed by measuring neurosphere’s diameter^57^. Briefly, 5000 living cells were seeded per well in 24-well plates and cultured for up to 96 h to evaluate the expansion rates. Size of 100 neurospheres (expressed as neurospheres diameters) was measured in three independent experiments. Images were taken with a microscope Olympus BH-2 and analysed using the freeware image J (National Institutes of Health, freeware).

### Liposome preparation

Concentrated lipid stocks were prepared as previously described^58^. Briefly, pure lipids were diluted in chloroform and dried in acid-washed glass centrifuge tubes under a stream of nitrogen. Phospholipid samples were suspended at 2–6 mM in phosphate-buffered saline at pH 7.2 and sonicated twice for 5 min at power setting 0.2–0.5% amplitude. All samples were sterilized with 0.22 μm-pore filters (Sartorius). The recovery of phospholipids after filtration was typically 90% or more.

### Immunocytochemistry

Cells were cultured on PDL (10 μg/ml)-coated glass coverslips in 24-well plates as previously described^15^. After different time of incubation, cells were fixed in 4% (w/v) paraformaldehyde-sucrose for 30 min at room temperature, permeabilized with 0.2% Triton X100 and blocked for 1 h in 5% BSA. Cells were incubated with the primary antibody overnight at 4 °C followed by incubation with the fluorescently labelled secondary antibody for 1 h at room temperature. Primary and secondary antibodies were diluted as follows: rabbit anti-βIII-tubulin (1:500), mouse anti-MAP2 (1/200), mouse anti-synaptophysin (1:300), rabbit anti-PSD95 (1:500), anti-rabbit Alexa Fluor® 488-labeled (1:500) and anti-mouse Cy3-labeled (1:300). To visualize nuclei, cells were counterstained and mounted with ProLong Gold antifade reagent containing DAPI (Molecular probes, Life technologies).

### Calcium imaging

Living-cell microscopy to detect depolarization-dependent calcium influx was performed as previously described^29^. A confocal laser system (Zeiss LSM 880) and the fluorescent calcium indicator Fluo-3 (Molecular Probes) were used to measure the intracellular free calcium concentration. Cells were loaded with the Ca^2+^-sensitive fluorescence indicator Fluo-3/AM (3 μM; Biotium) at 37 °C for 60 min in a Ringer buffer containing (in mM) 120 NaCl, 2.5 KCl, 20.0 Hepes (pH 7.4), and 11.0 glucose, and depolarized with high-KCl (100 mM) in Ringer solution as previously described. Fluorescence was measured every 0.25 s for a total of 12 s.

### Microscopy and image analysis

Micrographs were acquired using a confocal microscope (Zeiss LSM 880) or the Nikon Model Eclipse 800 microscope and quantitative analyses were performed with Image J” (NIH). Cells were counted from twenty randomly selected fields per well for each individual experiment. At least three independent experiments were performed. The percentage of neuronal cell population was calculated against the DAPI-positive total cell number which includes undifferentiated stem cells and differentiated neurons. Cells bearing at least one neurite equal or longer than the soma diameter were considered to be differentiated. Soma size, spines and number of synaptophysin or PSD-95 containing vesicles were measured and counted manually. To differentiate dystrophic and normal neuronal populations, 100 neurons from each experiment were manually selected and analysed. The fluorescence intensity was quantified using ImageJ and displayed in corrected total cell fluorescence (CTCF) = integrated density − (area of selected cell × mean fluorescence of background readings)^59^.

### Western Blot analysis

Western blot experiment was developed following protocols previously described^15,45^. Neurosphere-derived cells were plated at a density of 1.5 × 10^5^ and cultured on PDL-coated 35 mm culture dishes under differentiation conditions. Three days later, cells were collected, resuspended in lysis buffer (50 mM Tris–HCl pH 8.0, 50 mM KCl, 10 mM EDTA, Nonidet P-40 1%, 20 mM NaF, 1 mM Na_3_VO_4_, 1 mM PMSF and 1:1000 protease inhibitor cocktail) and sonicated five times at 5% amplitude for 5 s (Sonics and Materials Inc-Vibra CellTM). Protein concentration was determined using bovine serum albumin (BSA) as standard protein and “PierceTM BCA Protein Assay Kit (Thermo Scientific)”. 20 μg of cell lysate were resolved on 12% SDS-polyacrilamide gel electrophoresis (PAGE) and transferred to a nitrocellulose membrane (Amersham, GE Healthcare). After blocking overnight with 5% non-fat milk in 0.1% Tween TBS and washing, blots were incubated with anti-synaptophysin (1:300) and anti-PSD-95 (1/1500) overnight at 4 °C. Peroxidase-conjugated anti-mouse IgG (1:8000, Jackson Immuno Research) was used as secondary antibody. For loading protein control anti-β-Actin (1:6000) was used and developed with secondary antibody peroxidase-conjugated anti-mouse IgG (1:8000, Jackson Immuno Research). Labelled proteins were detected with chemiluminescence reagents (AmershamTM ECLTM Prime Western Blotting Detection Reagent, GE Healthcare).

### Statistical analysis

Data represent the mean value ± SEM of at least three independent experiments and each individual experiment was performed in technical triplicate. Statistical significance was determined by either Student’s t-test or One-Way ANOVA followed by Tukey's test using Prism (GraphPad Software Inc.). p-values lower than 0.05 were considered statistically significant.

## Supplementary Information

Supplementary Information 1.

Supplementary Video 1.

Supplementary Information 2.
