# Supplementary material for: Phosphatidylcholine restores neuronal plasticity of neural stem cells under inflammatory stress
Source: Sci Rep. 2021 Nov 24;11:22891. doi: 10.1038/s41598-021-02361-5 (PMC8613233; doi:10.1038/s41598-021-02361-5)
Supplement: Supplementary file 1 — Supplementary Information 1. [file 41598_2021_2361_MOESM1_ESM.pdf]

<u>Supplementary Info File</u>

Phosphatidylcholine restores neuronal plasticity of neural stem cells under inflammatory stress

Dario Magaquian, Susana Delgado Ocaña, Consuelo Perez and Claudia Banchio.

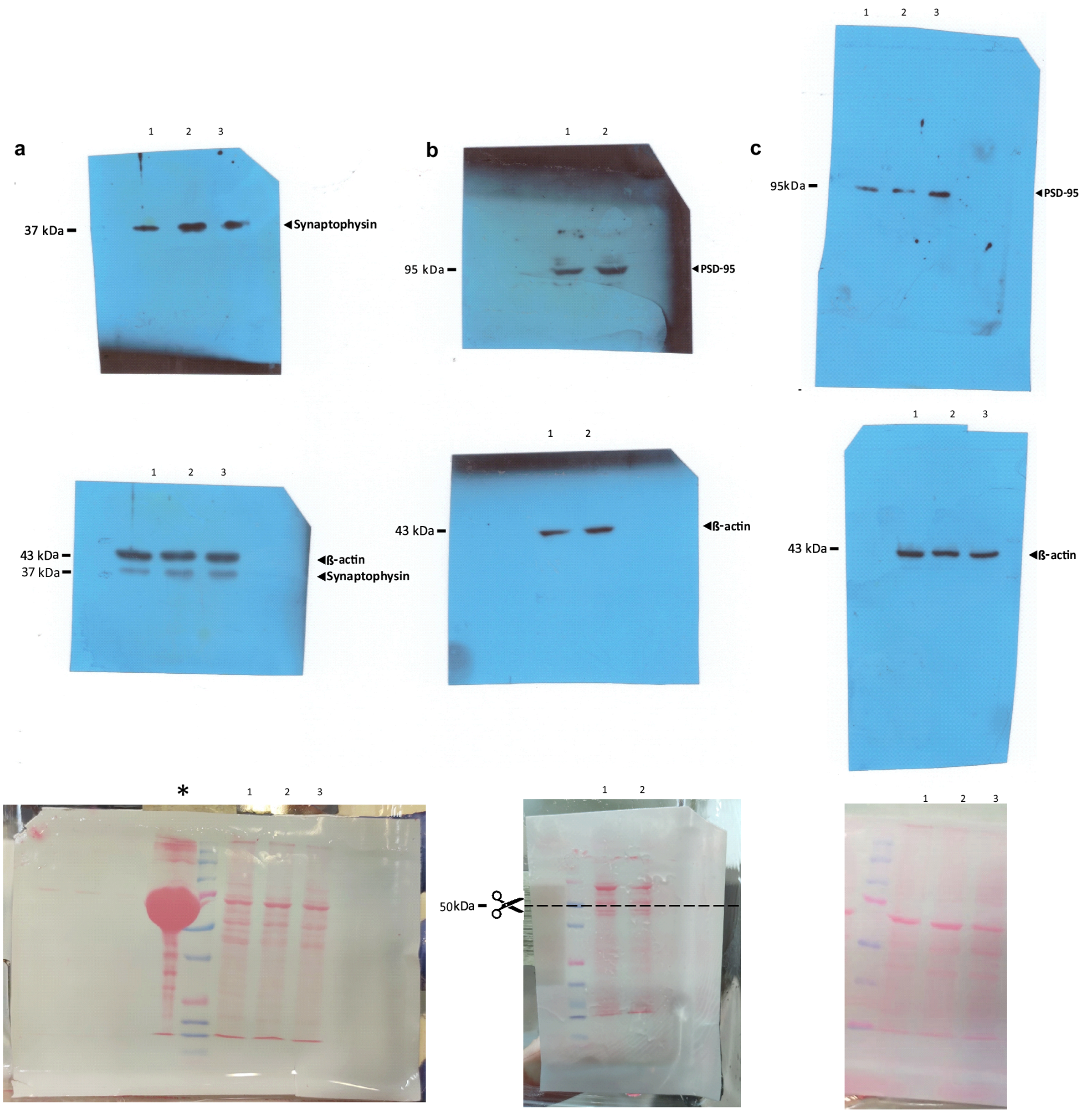

PtdCho supplementation restores synaptic defects caused by AM. a) Western Blots against Synaptophysin in NSCs exposed to AM (20% V/V) (Line 1) or in the presence of PtdCho (50 µM) (Line 2) or control (Line 3). We loaded 20 ug of proteins in a 12.5% SDS PAGE, transfer to nitrocellulose membrane (1.5 h/300mA) and blocked 1h in T-TBS milk 5%. We assayed with anti-synaptophysin (1/300 overnight at 4 °C) followed by HRP-mouse secondary antibody (1/10000) during 1h. We developed with Amersham ECL

Prime Western Blotting Detection Reagent and expose to Rx Amersham Hyperfilm ECL for 2 min. The membrane blocked ON in T-TBS milk 5% and incubated with and assayed as loading control anti-β-actin (1/6000 overnight at 4 °C) followed by HRP-mouse (1/10000) during 1 h. We developed with Amersham ECL Prime Western Blotting Detection Reagent and expose to Rx Amersham Hyperfilm ECL for 2 min. Representative image of membrane stained with Ponceau. **b and c)** Western blots against PSD-95 in NSCs exposed to 20% V/V of AM (Line 1), in the presence of PtdCho (50 μM) (Line 2) or under control (Line 3). We loaded 20 ug of proteins in a 12.5% SDS PAGE, transfer to nitrocellulose membrane (1.5 h/300mA) and blocked 1h in T-TBS milk 5%. We assayed with anti-PSD95 (1/1500) or anti-β-actin (1/6000) overnight at 4 °C, followed by HRP- mouse secondary antibody (1/10000) during 1h. We developed with Amersham ECL Prime Western Blotting Detection Reagent and expose to Rx Amersham Hyperfilm ECL for 2 min. Representative image of membrane stained with Ponceau. * unrelated sample.
