# Supplementary material for: Phosphatidylcholine restores neuronal plasticity of neural stem cells under inflammatory stress
Source: Sci Rep. 2021 Nov 24;11:22891. doi: 10.1038/s41598-021-02361-5 (PMC8613233; doi:10.1038/s41598-021-02361-5)
Supplement: Supplementary file 3 — Supplementary Information 2. [file 41598_2021_2361_MOESM3_ESM.pdf]

Phosphatidylcholine restores neuronal plasticity of neural stem cells under inflammatory stress

Dario Magaquian, Susana Delgado Ocaña, Consuelo Perez and Claudia Banchio*

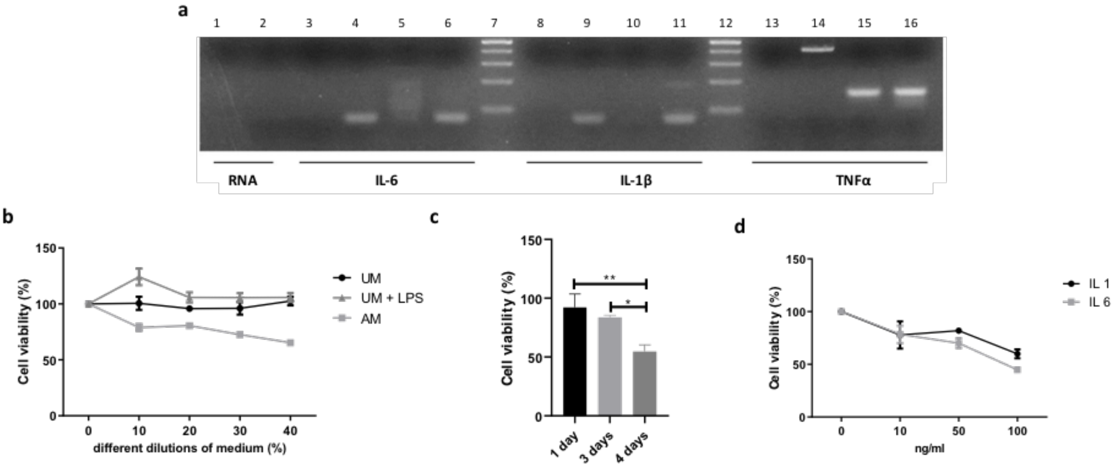

Supplementary figure 1 - Characterization of macrophages-activated media. a) Representative image of the amplification products obtained after the RT-PCR made from total RNA of Raw 264.7 cells grown in DMEM 10% SFB in the presence of 1 μg/ml LPS for 18 hours (n = 2). -LPS: RNA isolated from unstimulated Raw 264.7 cells and treated with RQ1 DNase was used as a template of the PCR reaction to control for the presence of traces of genomic DNA. +LPS: RNA isolated from LPS-stimulated Raw 264.7 cells and treated with RQ1 DNase was used as a template of the PCR reaction to control for the presence of traces of genomic DNA. C-: PCR water control (negative control). C+: PCR positive control (mouse genomic DNA). -cDNA: PCR product from total cDNA generated from unstimulated Raw 264.7 cells. +cDNA: PCR product from total cDNA generated from LPS-stimulated Raw 264.7 cells. MWM: Molecular weight marker, 100 bp. **b)** Cells were incubated with different dilutions of medium obtained from LPS-stimulated macrophages (AM), medium obtained from macrophages without activation as control medium (UM) or control medium with LPS (UM + LPS) for a period of 4 days, the viability of the NSCs was analysed by MTT assay. **c)** Cells were incubated with 20% of LPS-stimulated macrophages (AM) for the indicated periods of time, and the viability of the NSCs was analysed by MTT assay**.** Data were presented as mean ± SEM.**p<0.05. **d)** After incubating the cells with the indicated concentrations of interleukins for a period of 3 days, the viability of the NSCs was analysed by MTT assay.

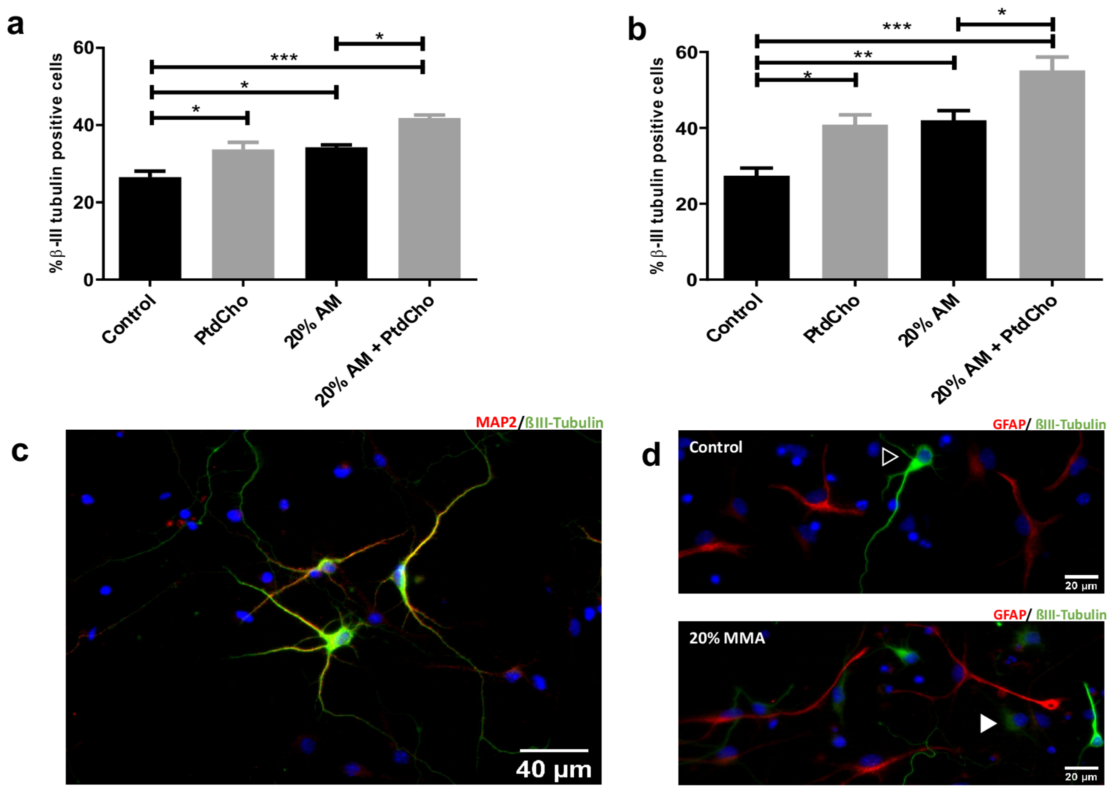

Supplementary figure 2- NSCs differentiation is affected by inflammation and restored by PtdCho. Percentage of β-III Tubulin positive cells analysed by immunocytochemistry coupled to fluorescence microscopy of NSCs exposed to 20% V/V of AM and UM in the presence or in the absence of PC 50 µM during 1 **(a)** and 2 **(b)** days. Graph is representative of three independent experiments. Data were presented as mean ± SEM. ***$p < 0.001$; **$p < 0.01$; *$p < 0.05$. **c)** Representative images of β-III Tubulin and MAP-2 positive neurons incubated during 3 days under control condition (100X). **d)** Representative images of β-III Tubulin and GFAP positive cells incubated during 3 days under control or 20% V/V of AM conditions. Open arrow indicates a normal neuron and white arrow dystrophic neurons.

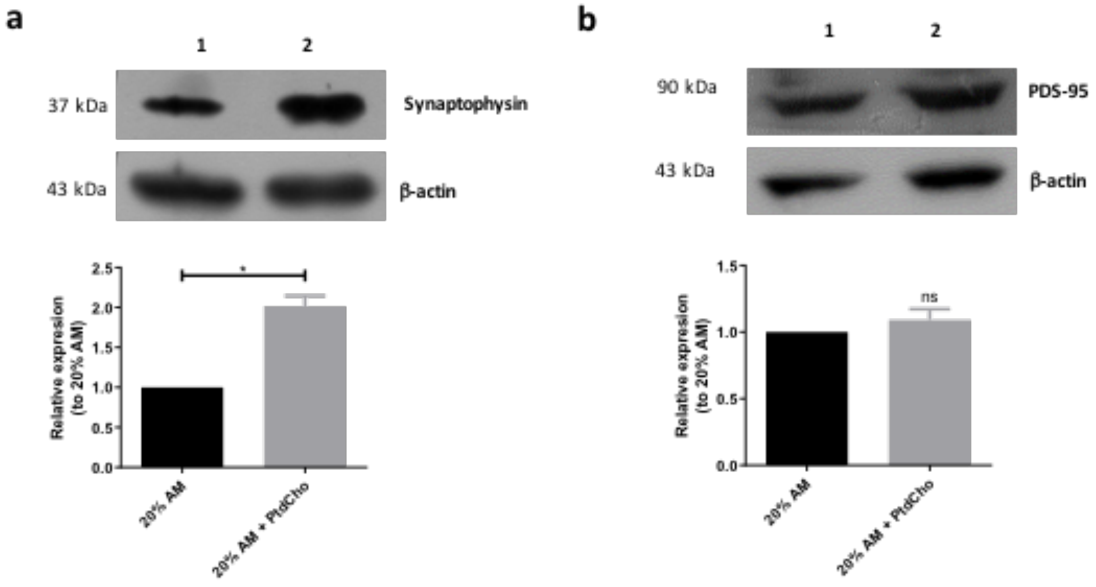

Supplementary figure 3- Synaptophysin and PSD-95 levels under inflammation and PtdCho treatment. Representative image of Western Blot showing Synaptophysin **(a)** and PSD-95 **(b)** levels in NSCs exposed to 20% V/V of AM in the presence or in the absence of PtdCho (50 μM) during 3 days. β-actin was used as control loading. The gels/blots displayed here are cropped, and without high-contrast (overexposure). The full-length gels and blots are included in a Supplementary Information file. Densitometric analysis, *$p < 0.05$, ns: no statistical significance (Student's T-test).

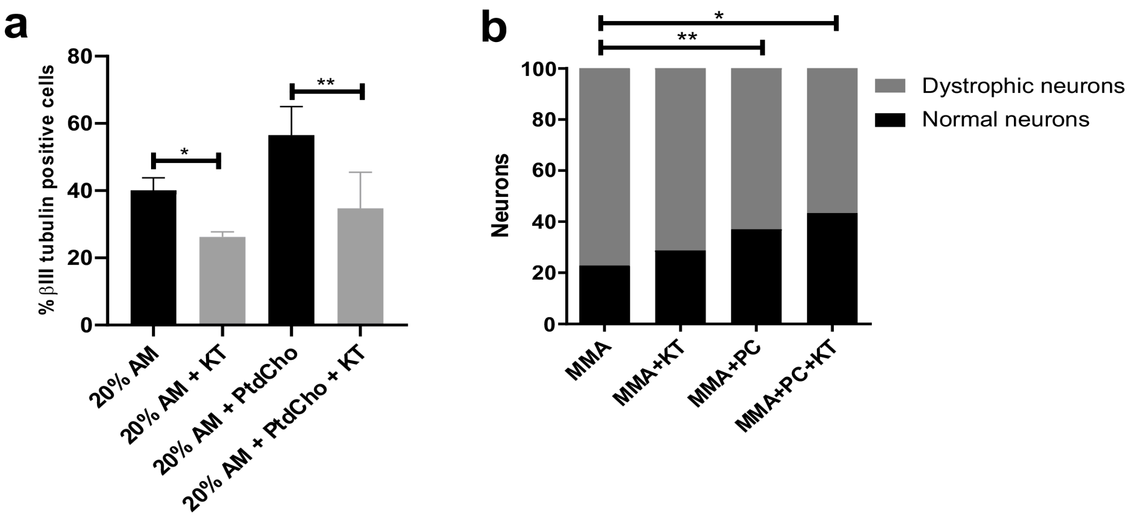

Supplementary figure 4 - PtdCho effects and the PKA/CREB signalling pathway under inflammatory conditions (AM). a) Percentage of β-III Tubulin positive cells analysed by immunocytochemistry coupled to fluorescence microscopy of NSCs exposed to 20% V/V of AM in the presence or in the absence of PC 50 μM and PKA inhibitor (KT5720 (10 μM)) after 72 hours. Cells were incubated during 30 min with the PKA inhibitor prior to liposomes addition. Graph is representative of three independent experiments. Data were presented as mean ± SEM. ***$p < 0.001$; **$p < 0.01$; *$p < 0.05$. **b)** Number of normal and dystrophic neurons under inflammatory conditions in the presence or in the absence of PtdCho or KT5720, **$p < 0.05$ ***$p < 0.001$ (Student's T-test).

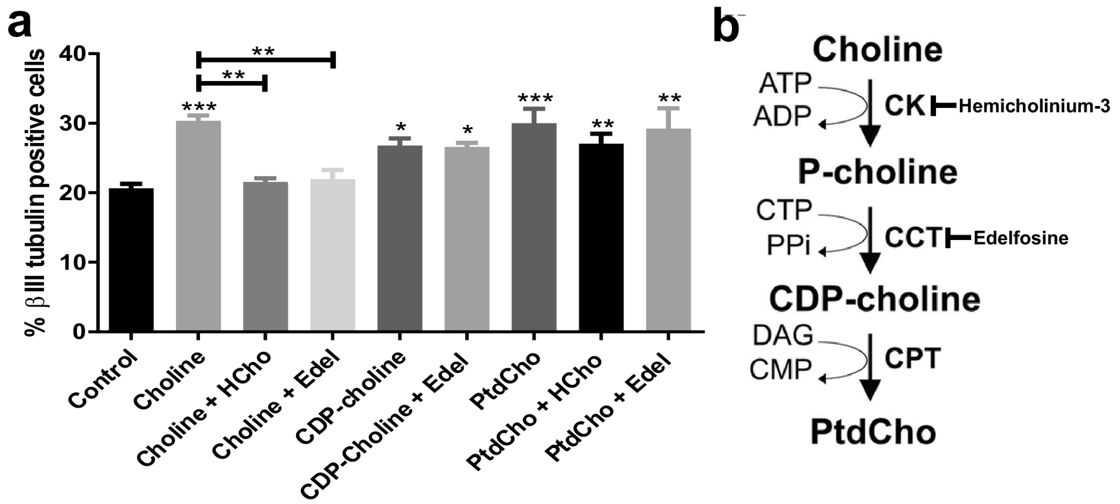

Supplementary figure 5 – Effect of choline, CDP-choline and PtdCho on neuronal differentiation. a) Percentage of β-IIITubulin positive cells analysed by immunocytochemistry coupled to fluorescence microscopy of NSCs treated during 24 hours with choline (50 µM), CDP-choline (50 µM), PtdCho (50 µM) in the presence or in the absence of the indicated inhibitors hemicholineum-3 (HCho, 50 µM) for choline kinase and Edelfosine (Edel, 5 µM) for phosphocholine cytidylyltransferase α. Cells were incubated during 30 min with the inhibitors prior to liposomes addition. Graph represents the percentage of neuronal differentiation measured in three independent experiments. Data were presented as mean ± SEM. ***$p < 0.001$; **$p < 0.01$; *$p < 0.05$. **b)** Schematic representation of the Kennedy pathway for PtdCho biosynthesis.

Supplementary Movie: Real-time observation of calcium influx after KCl. Cells were loaded with the Ca2+-sensitive fluorescence indicator Fluo-3/AM and depolarized with high-KCl (100 mM) in Ringer solution. Fluorescence was measured every 0.25 s for a total of 12 s with confocal laser system (Zeiss LSM 880). Assay condition are indicated in the video.
